# Genetic Modifying Factors of Cystic Fibrosis Phenotype: A Challenge for Modern Medicine

**DOI:** 10.3390/jcm10245821

**Published:** 2021-12-13

**Authors:** Lăcrămioara Ionela Butnariu, Elena Țarcă, Elena Cojocaru, Cristina Rusu, Ștefana Maria Moisă, Maria-Magdalena Leon Constantin, Eusebiu Vlad Gorduza, Laura Mihaela Trandafir

**Affiliations:** 1 Department of Medical Genetics, Faculty of Medicine, “Grigore T. Popa” University of Medicine and Pharmacy, 700115 Iasi, Romania; ionela.butnariu@umfiasi.ro (L.I.B.); abcrusu@gmail.com (C.R.); vgord@mail.com (E.V.G.); 2 Department of Surgery II—Pediatric Surgery, “Grigore T. Popa” University of Medicine and Pharmacy, 700115 Iaşi, Romania; 3 Department of Morphofunctional Sciences I, “Grigore T. Popa” University of Medicine and Pharmacy, 700115 Iaşi, Romania; 4 Department of Mother and Child, Faculty of Medicine, “Grigore T. Popa” University of Medicine and Pharmacy, 700115 Iasi, Romania; stefana-maria.moisa@umfiasi.ro (Ș.M.M.); laura.trandafir@umfiasi.ro (L.M.T.); 5 Medical I Department, “Grigore T. Popa” University of Medicine and Pharmacy, 700115 Iasi, Romania; leon_mariamagdalena@yahoo.com

**Keywords:** cystic fibrosis, CFTR, modifier genes, phenotypic variability, GWAS

## Abstract

Cystic fibrosis (CF) is a monogenic autosomal recessive disease caused by cystic fibrosis transmembrane conductance regulator (*CFTR*) gene mutations. CF is characterized by a high phenotypic variability present even in patients with the same genotype. This is due to the intervention of modifier genes that interact with both the CFTR gene and environmental factors. The purpose of this review is to highlight the role of non-CFTR genetic factors (modifier genes) that contribute to phenotypic variability in CF. We analyzed literature data starting with candidate gene studies and continuing with extensive studies, such as genome-wide association studies (GWAS) and whole exome sequencing (WES). The results of both types of studies revealed that the number of modifier genes in CF patients is impressive. Their identification offers a new perspective on the pathophysiological mechanisms of the disease, paving the way for the understanding of other genetic disorders. In conclusion, in the future, genetic analysis, such as GWAS and WES, should be performed routinely. A challenge for future research is to integrate their results in the process of developing new classes of drugs, with a goal to improve the prognosis, increase life expectancy, and enhance quality of life among CF patients.

## 1. Introduction

Cystic fibrosis (CF) is a life-shortening and multisystem autosomal recessive disease, caused by mutation of transmembrane conductance regulator (CFTR) gene that encodes for a chloride channel expressed on the membrane of epithelial cells of the respiratory tract, intestine, hepatobiliary system, and exocrine sweat glands [1,2]. The disease affects about 1 in 2500 people of European descents, is less common among African Americans and Hispanics, and is rare among Asians [1,2]. One in twenty-five people of European descent is a healthy carrier of a *CFTR* gene mutation [1,2,3].

The diagnosis of CF is primarily based on abnormal CFTR function obtained through sweat chloride testing (≥60 mmol/L) associate with a positive newborn screening, clinical features consistent with CF, or a positive family history [1,2].

Pulmonary manifestation is the main cause of mortality and morbidity because airways obstruction by abnormally thick mucus and chronic bacterial infections can cause a persistent inflammatory response, which developes bronchiectasis and pulmonary emphysema evolving over time to respiratory failure [4,5,6]. Most patients have chronic sinusitis and/or nasal polyposis, mostly asymptomatic. Intestinal obstruction in the neonatal period (meconium ileus) (MI) is a complication present in approximately 15% of newborns with CF [2]. The presence of cystic fibrosis-associated liver disease (CFLD) in 30% of cases is correlated with a reduced lifespan, due to severe liver cirrhosis and portal hypertension [2,6].

Many patients with CF have pancreatic damage manifested by severe exocrine pancreatic insufficiency (PI), which requires enzyme replacement therapy. Diabetes mellitus is present in about 2% of children, 19% of adolescents, and 40% of adults with CF [2]. The presence of CF-related diabetes (CFRD) was correlated with more severe lung disease and decreased longevity [5,6,7].

In recent years, the life expectancy of CF patients has significantly improved (from less than one year to over 40 years), due to specific therapies and therapy with pancreatic enzymes supplement, mucus thinners, bronchodilators, antibiotics, aerobic exercise, and autogenous drainage of bronchial secretions, which indicates an important effect of non-genetic environmental factors on phenotypic variability of the disease [2,6]. Based on 2019 Cystic Fibrosis Foundation Patient Registry data, the life expectancy of people with CF who are born between 2015 and 2019 is predicted to be 46 years. It is expected that half of the children with CF born in 2019 will live until the age of 48 [1].

The lethal forms of the disease are determined by severe dysfunction of the CFTR protein and are associated with chronic lung disease progressing to respiratory failure. Apart from these, there is a distinct spectrum of clinical manifestations, nonlethal, caused by mutations in the *CFTR* gene that cause CFTR protein dysfunction, but which do not meet the diagnostic criteria for cystic fibrosis, called CFTR-related disorders [1]. The term CF-related metabolic syndrome (CRMS) is used in asymptomatic infants with elevated trypsinogen level in newborn screening, associated with sweat chloride values of ≤60 mEq/L and to whom one or two mutations in the *CFTR* gene are detected, of which at least one is not classified as a disease-causing pathogenic variant. Therefore, the diagnostic criteria for CF are not met [1,2].

Although CF is a monogenic disease, with Mendelian transmission, it is characterized by a high phenotypic variability, being specific to the organ, determined both by the great diversity of mutations of the *CFTR* gene and by the interaction with other genes, called modifier genes, or with environmental factors [7].

The aim of the paper is to make a review of literature data about the factors that may influence the phenotype of patients with cystic fibrosis, providing a perspective on the role of modifier genes intervention (epistasis), without minimizing the role of environmental factors. The methods approached over time for modifier gene studies are also presented, taking into account both the results obtained and the advantages and limitations of each type of study, as well the perspectives that new information obtained opens up for future research related to development of new classes of drugs, which is much more effective.

## 2. Literature Search Strategies and Data Collection

The data synthesized and presented in this review were obtained by searching the databases (literature), using the following keywords: cystic fibrosis, CFTR, modifier genes, phenotypic variability in CF, candidate gene studies, genome-wide association studies (GWAS) or whole exome sequencing (WES), and CF-associated comorbidities (CF lung disease, meconium ileus (MI), CF-associated liver disease (CFLD), pancreatic insufficiency (PI), and CF-related diabetes (CFRD)) (Table 1).

## 3. The Role of Genetic Heterogeneity in Cystic Fibrosis and Genotype-Phenotype Correlation

Identification and cloning of the cystic fibrosis gene (*CFTR)*, located on chromosome 7q31.2p, as well as molecular research and identification of the most common types of mutations, facilitated major advances in medical genetics. This has since improved understanding of the pathophysiological mechanisms of the disease and provided the premises for effective targeted therapy. More than 2000 mutations are currently identified (Cystic Fibrosis Gene Analysis Consortium, www.genet.sickkids.on.ca/cftr/, accessed on 19 Nobember 2021), located throughout the *CFTR* gene [7,69]. Approximately 1200 of these are considered pathogenic (disease-causing mutations), while other alleles have an uncertain pathogenic significance or are considered non-pathogenic (non-disease-causing mutations). Despite the increased allelic heterogeneity, the most common mutation detected worldwide in over 70% of patients with CF consists of the deletion of phenylalanine at position 508 of amino acids chain (F508del). The F508del mutation is detected more frequently in the European population (Caucasians), in the Mediterranean area, and in Northern Europe, but the percentage varies in different ethnic groups [6].

Affected individuals are frequently homozygous for the F508del mutation (identical allele genes) or compound heterozygotes (with two different *CFTR* gene mutations), while carriers of a single abnormal allele are asymptomatic. Two healthy carrier heterozygous parents have a 25% risk of having an affected newborn, with the disease having an autosomal recessive transmission [1,2]. The *CFTR* gene contains 27 exons and encodes a protein with 1480 amino acids that acts as a transmembrane chlorine channel (controlled by cAMP) and regulator of other ion channels (including the transport of Na^+^ ions in the airways) [79,80,81].

The CFTR protein is responsible for regulating the transport of ions and fluids in epithelial cell membranes, especially in the pulmonary airways and pancreatic ducts. CFTR protein dysfunction causes changes in the physiological transport of ions in cell surfaces with the production of increased viscosity mucus in the bronchial tract and digestive tract (pancreas, liver, and biliary tract), leading to obstruction and compromise defense mechanisms, which ultimately leads to tissues destruction [79].

The *CFTR* gene mutations are classified into six classes, depending on their pathogenic role at the molecular level and impact on CFTR protein function (Table 2) [5,69,82,83,84].

**Figure 1:**
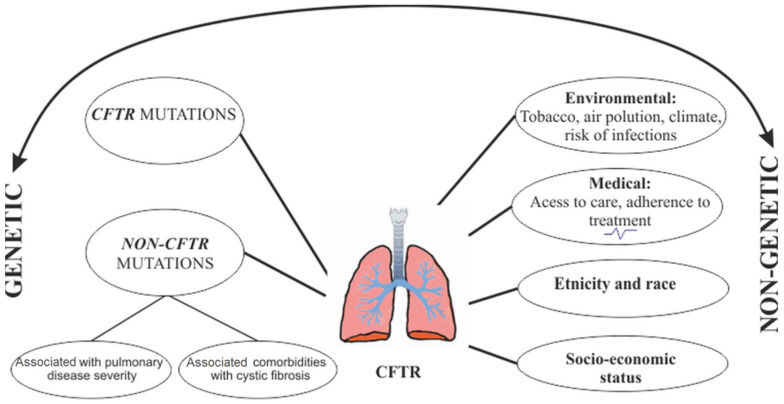
Genetic and non-genetic modifiers of phenotype in cystic fibrosis: the interaction between the CFTR genotype with modifier genes and environmental factors.

The different classes of *CFTR* mutations are associated with phenotypic variations, especially in the severity of clinical manifestations, correlated with increased morbidity and mortality, as well as with responses to different types of therapies [3,5,6,7]. However, the phenotypic effect on mutations is strongly influenced by environmental factors (passive smoking, socio-economic status, access to health care, etc.) and by the interaction with multiple genes (epistasis), especially with regard to CF lung diseases [79]. Epigenetic changes (DNA methylation, histone protein modification) are also discussed [2]. The factors that modify the phenotype in CF are shown in Figure 1.

## 4. The Role of Modifier Genes and Phenotypic Variability in Cystic Fibrosis

### 4.1. The Concept of Modifier Genes

The observation that people (from the same family or unrelated) who have the same mutation of CFTR may have variable clinical manifestations, both in terms of the severity of pulmonary manifestations and associated comorbidities, was discussed in relation to the existence of the other phenotype modifying factors (non-CFTR) classified into genetic and non-genetic (Figure 1). In the early 1990s, there was evidence that, although the CFTR mutation class was a fairly good predictor of pancreatic disease associated with CF, this was not the case with CF lung disease.

The relatively high incidence of CF has made it possible to conduct numerous heritability studies [85]. The potential role of modifier genes in CF has been demonstrated by studies in twins, which revealed a higher concordance of the phenotype in monozygotic twins (MZ) compared to dizygotic twins (DZ). Some studies have suggested the possibiity that more than 50% of the variation in the lung phenotype was due to the intervention of modifier genes [69,86]. Other modifiers of the response to various therapies will be discovered in the next period, based on increasing access to therapies that restore CFTR protein function [69].

**Figure 2:**
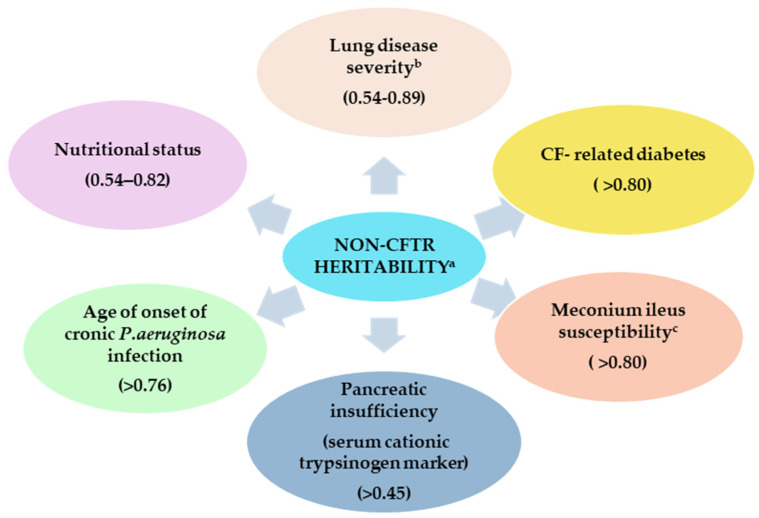
Heritability estimates for cystic fibrosis related phenotypes established from twins and sibling studies [74,85,87,88,89,90,91,92]. ^a^ refers to the heritability that is not due to differences in phenotype that occur across different *CFTR* mutations; ^b^ depending on the method used for evaluation; ^c^ in CF patients with two severe mutations and pancreatic insufficiency.

Various studies have shown a high degree of heritability for several CF phenotypes, including pulmonary manifestations (0.54–0.80) [85,87,88], meconium ileus (>0.80) [89], early exocrine pancreatic insufficiency (>0.45) [90], nutritional status (body mass index) (0.54–0.82) [91], age of onset of chronic *Pseudomonas aeruginosa* infection (>0.76) [92], and CF-related diabetes (CFRD) (>0.80) [74]. All these vary depending on the specific analyses and the specific population studied (dizygotic versus monozygotic twins); however, they indicated that non-CFTR modifiers play an important role in disease-specific manifestations (Figure 2).

### 4.2. The Role of Modifier Factors

Previous studies have indicated the existence of a strong influence of genetic modifying factors on CF phenotype [2,93]. The identification of macromolecular complexes and deciphering the mechanisms, which can be controlled by intracellular trafficking and CFTR activity [2,94,95], acted as the basis for further studies, based on candidate gene analysis and extensive genome-wide analysis (GWAS). Two types of approaches were used to identify CF modifiers: case-control association studies and family-based linkage studies. Both methods have advantages and disadvantages, for example the high difficulty of detecting common genetic variants (for case-control association studies) or a better differentiation between the effects caused by environmental factors and those caused by genetic factors (in the case of family studies). Therefore, complementary studies using both methodologies are recommended [2,79,80]. Furthermore, any association between a possible candidate chromosomal region, a specific locus, or a single gene variant should be confirmed in other patients, individually and in separate control cohorts. The results of the studies can be influenced by ethnicity, environmental factors, treatment, and socio-economic status [2,79].

**Figure 3:**
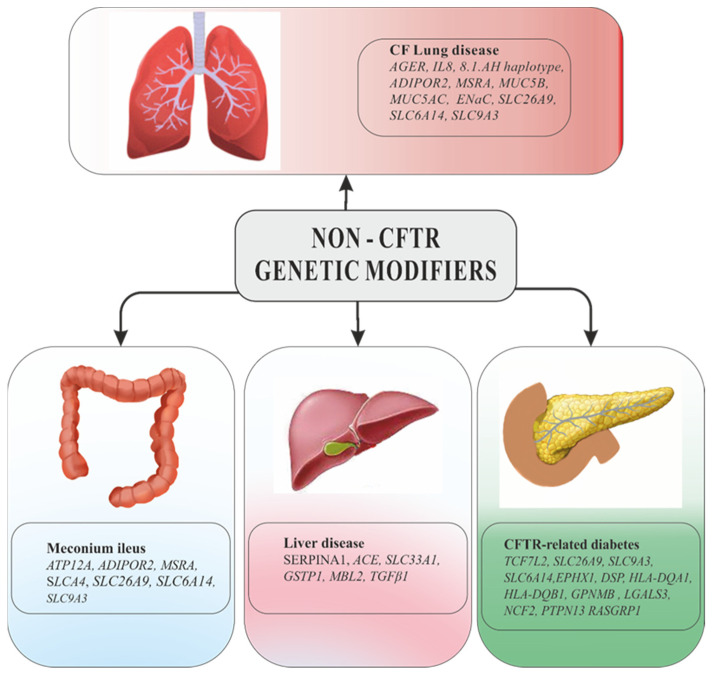
Possible candidate genes that modify the pulmonary phenotype and comorbidities associated with cystic fibrosis.

As more and more possible candidate genes were discussed and larger cohorts were analyzed, was made the transition to GWAS or WES [2,96]. In this sense, effort is still made by the International CF Gene Modifier Consortium (CGM), which analyzed samples of patients from the United States, Canada, and France [2,97,98]. Initially, the study focused on the modifying factors of the pulmonary phenotype, later expanding to the research on other manifestations associated with CF (meconium ileus and diabetes) [97,98]. Possible candidate genes which modify pulmonary phenotype and CF-associated comorbidities are presented in Figure 3.

In the next section, we will present a summary of data from the literature that consists of arguments that phenotypic variability in patients with CF significantly depends on interactions with other genetic and non-genetic (environmental) factors.

## 5. Candidate Gene Studies

### 5.1. Modifier Genes of CF Lung Disease

Lung disease is the main source of morbidity and mortality in CF [7,69,85,96,97,98,99,100]. Allelic variants of *CFTR* mutations do not explain the wide variation in the severity of lung disease [101,102]; however, studies on twins and siblings show substantial heritability (h > 0.5), highlighting differences that may exist when performing lung function measurements in CF patients (Figure 2) [88].

The results of candidate gene studies have been contradictory, with limited large-scale reproduction, suggesting that they represent a small proportion of hereditary variation in lung function in CF. The identification of other genetic modifiers could identify potential mechanisms that cause variations in lung function in CF, but also for other common diseases, such as chronic obstructive pulmonary disease (COPD), and would suggest new targets for therapeutic intervention. GWAS offer the possibility of identifying new loci and, implicitly, new modifier genes in monogenic diseases, such as CF [98].

Pulmonary manifestations in CF are due to inflammation of the lower respiratory tract with mucus accumulation and recurrent chronic infection [103]. The severity of pulmonary manifestations is extremely variable, even in patients of the same age and sex, as well as those who have the same genotype and receive the same treatment [88]. Lung function is the main marker for assessing CF severity. The most commonly used biomarker is forced expiratory volume in the first second (FEV1) [88,104,105,106].

**Figure 4:**
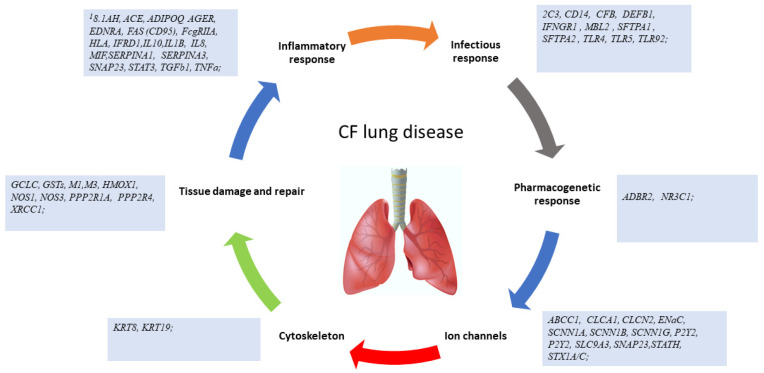
Pathogenic mechanisms correlated with the severity of CF lung disease.

The mechanisms by which modifier genes act in CF lung disease involve numerous molecules which intervene in the host’s inflammatory and defense response to infectious agents, drug response, ion transport to the cell surface (ion transport channels, including CFTR), repair mechanisms of lung injuries, but also many epigenetic mechanisms (DNA methylation) and ribosomal proteins (Figure 4) [7,8,9,11,14,15,16,69,79,80,81,86,106].

#### 5.1.1. Candidate Genes Related to the Inflammatory Mechanism

a. Cytokines

*Transforming Growth Factor β 1 (TGFβ1)*

TGFβ1 is part of the family of factors involved in cell growth and differentiation, and is a multifunctional cytokine, encoded by a gene located on chromosome 19q13.1-q13.3. Elevated levels of TGFβ1 have been identified in bronchoalveolar lavage fluid of patients with severe CF lung disease [8,9,10,11]. Extensive studies, including one based on the concept of candidate genes, have demonstrated the role of TGFβ1 in the occurrence of pulmonary fibrosis, secondary to the inflammatory response. Different allelic variants of the TGFβ1 gene have been identified in patients with pulmonary fibrosis and asthma, and may cause chronic obstructive pulmonary disease (COPD) in interaction with environmental factors (cigarette smoke) [8,12,13].

The most studied remain the alleles located at the level of the C−509T promoter and at the level of exon 1, codon 10–869 T/C (Leu10/Pro10), and 915 G/C (Arg25/Pro25) associated, in some studies with a reduced lung function, aspect unconfirmed by other studies (in which other alleles involved were identified) [8,9]. Although contradictory, the results of numerous studies have suggested that the *TGFB1* gene is an important modifier of lung function, and existing phenotypic variations are correlated with the presence of ancestral allelic variants or with interaction with environmental factors (exposure to cigarette smoke) [14,15].

*Interleukine 8 (IL8)*

Interleukin 8 (CXCL8) is a member of the CXC family of chemokines, encoded by a gene located on chromosome 4q13.3. IL8 is a potent neutrophilic chemoattractor that intervenes in the inflammatory response of the lungs. A study by Hillian et al. [16] identified three polymorphic variants of the *IL8* gene that are associated with the severity of lung disease in CF: rs4073 (IL8 −251 T/A), rs2227306 (IL8 781 C/T), and rs2227307 (IL8 396 T/G) [16]. The same association would have a significance in favoring and maintaining the bacterial infection of the respiratory tract with *P. aeruginosa* [17].

*Interleukine 1 B (IL1B)*

The *IL1B* gene, located on chromosome 2q13, encodes a cytokine which is primarily produced by monocytes and mediates the acute phase response. In a 2009 study that included 808 patients with CF, Levy et al. [18] reported an association between allelic variants rs1143634 (exon 5) and rs1143639 (intron 6) and severe lung disease with respiratory dysfunction, in the absence of colonization with *P. aeruginosa*, present in many patients with CF [18]. These results were subsequently confirmed by a European family study by Labenski et al. [19].

*Interleukin 10*

The *IL10* gene, located on chromosome 1q32, encodes a cytokine with anti-inflammatory properties that plays a central role in limiting the host’s immune response to pathogens, thus playing an important role in CF lung disease [20,21]. Certain allelic polymorphic variants in the gene promoter are associated with elevated or decreased IL10 values. The allelic variant 1082G/A (rs1800896) causes an increased level of IL10 detected in patients who had airway colonization with *A. fumigatus* (allergic bronchopulmonary aspergillosis-ABPA), compared to those without infection. This could not be proven in patients with *P. aeruginosa* [17,22].

*Tumor Necrosis Factor Alpha (TNFα)*

The *TNFα* gene, located on chromosome 6p21.3, encodes a pro-inflammatory cytokine secreted by macrophages, monocytes, and airway epithelial cells during acute inflammation, and contributes to the exaggerated neutrophil-mediated inflammatory response, leading to necrosis or apoptosis. Several polymorphic variants of the *TNFα* gene present in several respiratory diseases (asthma and COPD) were studied, both at the level of the promoter (851 C/T, 308 G/A, 238 G/A) and at the level of intron 1 (691 G ins/del), producing variable results [23,24]. Kaluza et al. [25] demonstrated that the 308 G/A allelic variant modulates TNF production [25]. In another study, Hull et al. [86] provided evidence for an association between TNF polymorphism (TNFα −308 G/A allele) and the severity of phenotypic manifestations in CF [86]. Yarden et al. [26] analyzed four polymorphic variants of *TNFα* in a study that included 180 CF patients (from Belgium and the Czech Republic) with homozygous genotype F508del [26]. The allelic variant TNFα-308 G/A was most associated with proper lung function and late *P. aeruginosa* infection, and the allelic variants +691 G ins/del and 2851 C/T were associated with severe manifestations of lung disease [25,26].

b. Other Genes Involved in Inflammation

*The HLA System*

Genes encoding HLA antigens are located on chromosome 6p21. The ancestral haplotype 8.1 (8.1 AH) (known as the HLA haplotype A1-B8-DR3-DQ2), widespread in Caucasians, is associated with the TNF-mediated inflammatory response. Laki et al. [27] showed that, in CF patients with haplotype 8.1 AH, the onset of respiratory colonization with *S. aureus* and *P. aeruginosa* was late [27]. Carriers of the 8.1 AH haplotype have a significantly weaker lung function, with—6.4% of FEV1 compared to those who do not have this variant [28]. In CF as well as in asthma, the HLA-DR4 and DR7 alleles have been strongly associated with pulmonary aspergillosis (ABPA) [60]. The DR7/DQA * 0201 haplotype has been associated with elevated IgE levels and increased frequency of *P. aeruginosa* colonization in CF patients [29,30,31].

*Plasma Serine Protease Inhibitors Alpha-1 Antitrypsine (AAT)*

Alpha-1 antitrypine (AAT), a major inhibitor of plasma serine protease, is encoded by the *SERPINA1* gene, located on chromosome 14q32.13. AAT inhibits neutrophil elastase, which degrades elastin in the alveolar walls. In CF, AAT deficiency is associated with elevated levels of neutrophil elastase. Pulmonary AAT deficiency is also associated with other lung diseases, such as emphysema, asthma, and/or pulmonary bronchiectasis [8,32,33]. Morgan et al. [34] analyzed three allelic variants of the *SERPINA1* gene present in CF patients: Z and S allele deficiency (associated with decreased plasma AAT levels) and 1237 A/G variant (located in the 3 ‘amplifier region) associated with IL6-mediated AAT level regulation during infections [34]. However, the studies that analyzed the most numerous cohorts did not find any association of these three variants with the severity of lung disease in CF [9,35].

*AGER Gene*

The *AGER* gene encodes a receptor for advanced glycation endproducts (RAGE), a pro-inflammatory pattern recognition receptor (PRR) regarded as a central mediator in chronic inflammation and immune respons against pathogens. RAGE and its ligands are highly expressed in pulmonary tissue. Chronic activation of RAGE and increased airway inflammation lead to decreased lung function in CF patients [8,36]. A French study that included a cohort of 967 patients with the homozygous F508del genotype revealed a correlation between the presence of the 429 T/C allelic variant located in the AGER gene promoter and the severity of lung disease in CF [36].

*Macrophage Migration Inhibitor (MIF)*

MIF is a key pro-inflammatory mediator that contributes to the production of an excessive inflammatory response, either directly by inducing the secretion of pro-inflammatory cytokines or indirectly by its ability to overcome the anti-inflammatory effect of glucocorticoid. A polymorphism of the *MIH* gene represented by the presence of five CATT repetition at position 794 in the promoter region was associated with decreased gene expression [37]. Patients with CF with a single 5-CATT repeat allele had a low incidence of *P. aeruginosa* colonization and mild pulmonary dysfunction [38].

*Mucins*

Mucins, encoded by *MUC* genes, belong to a class of 18 proteins, which play an important role in the elimination of mucociliary secretions (especially *MUC5AC* and *MUC5B*). The presence of single nucleotide polymorphism (SNP) in the *MUC5B* gene promoter region (rs35705950) is considered the strongest risk allele associated with pulmonary fibrosis [39]. In a study of 762 patients, a correlation was found between the variable number of tandem repeats (VNTR) of the *MUC5AC* gene and the severity of lung disease, while 6.4 kb VNTR was associated with more severe pumonary disease [40,41].

#### 5.1.2. Candidate Genes Related to the Infectious Response

*Mannose-Binding Lectin 2 (MBL2)*

Mannose-binding lectin (MBL) is a synthesized liver protein that accumulates in the lungs during acute inflammation. MBL is encoded by the *MBL2* gene located on chromosome 10q11.2-q21 and plays a major role in infectious diseases, binding to bacteria (*S. aureus* and *P. aeruginosa*) [42]. *MBL2* gene polymorphisms located in the exon 1 and promoter region that are in strong linkage disequilibrium are responsible for eight haplotypes associated with low MBL serum levels. There are three mutations located in exon 1 which corresponds to the allelic variants MBL2 *D (Arg52Cys), B (Gly54Asp), and C (Gly57Glu), and that are responsible for decreased MBL function. These variants are commonly called the O alleles, while the wild-type allele that produces normal levels of protein is called MBL2 *A [42,43]. Mutant proteins have an abnormal structure and a short lifespan. People with a homozygous (O/O) genotype have extremely low levels of MBL protein, while the heterozygous genotype is associated with the presence of residual levels of protein [42]. Most studies have found an association between low MBL levels and an increased severity of lung disease, but there have also been studies that either showed no effect [9] or found less lung function in those with high protein production [43]. Some authors have stated that the adverse effect of insufficiency of *MBL2* alleles would be age-dependent, becoming evident at an age threshold appropriate to adolescence or puberty [42].

*Toll-like Receptors (TLRs)*

Toll-like receptors (TLRs) are pattern recognition receptors (PRRs) which play a crucial role in the initiation of innate immune response by detecting microbial infections and activating inflammatory responses. Microbial lipopolysaccharides (LPS) bind specifically to TLRs, which activate intracellular signaling and increase the expression of pro-inflammatory cytokines, evidence of association with chronic disease. Different polymorphic variants of the TLRs were analyzed, without proving a definite correlation with CF lung disease [44,45].

*CD14 Gene*

The *CD14* gene, located on chromosome 5q31.1, encodes a surface antigen that is preferentially expressed on monocytes/macrophages and acts as a receptor for lipopolysaccharides of Gram-negative bacteria (*P. aeruginosa*) [46]. Alexis et al. [47] showed that the presence of the 159 C/T allelic variant located in the *CD14* gene promoter is associated with a low level of CD14 in healthy children [47]. In a cohort of 105 CF patients compared to a control group, a higher frequency of the CD14—159 T/T genotype was observed among CF patients, without being associated with the severity of lung disease [48].

#### 5.1.3. Candidate Genes Involved in Epithelial Tissue Repair Mechanism

##### Glutathion and Glutathion-S-transferase

Glutathione (GSH) is a tripeptide that protects the lungs from damage caused by oxidants. In CF, CFTR deficiency induces low GSH transport, leading to systemic deficiency [107]. Several studies have identified an association between *GST* gene polymorphisms encoding glutathione-S-transferase and lung function. For example, in a study of 146 children with CF, those with a homozygous genotype for the GSTM3 * B allele had better lung function [108]. However, data on *GST* gene were somewhat contradictory. In one of the largest studies on the effect of *GST* variants on lung function in CF, no association was found with allelic variants *GSTM1*, *GSTP1*, or *GSTT1* [9].

##### Nitric Oxide Synthases (NOS)

Nitric oxide (NO), generated by NO synthase (NOS), is an important mediator of physiological processes in the airways and lung parenchyma, intervening in bronchodilation, inflammation, and repair of damaged tissues [49]. In the respiratory tract, NO is generated enzymatically by three distinct isoforms of NO synthase (NOS-1, NOS-2, and NOS-3). Unlike other inflammatory lung diseases (asthma, bronchiectasis), nitric oxide levels in expired air (FENO) are low in CF [50]. A polymorphism of AAT repeats in the *NOS* gene (located on chromosome 12q24) has been shown to be associated with a lower risk of colonization with *P. aeruginosa* and *A. fumigatus* [51] and a less severe course of lung disease in CF [52].

#### 5.1.4. Candidate Genes Associated with the Response to Drug Therapy

The β2 adrenergic receptors (ADRB2) are expressed on bronchial smooth muscle cells and mediate bronchodilation in response to exogenous and endogenous beta-adrenoceptor agonists. Single nucleotide polymorphism (SNP) in the *ADRB2* gene causes changes in amino acids (e.g., Arg16Gly and Gln27Glu) and, consequently, changes in receptor function. Studies on their involvement in the severity of CF lung disease are contradictory. In one of the studies, Hart et al. [53] did not find a relationship between Arg16Gly and Gln27Glu polymorphisms and the response to bronchodilators [53]. In another study, Marson et al. [54] concluded that people who have Gly or Glu allelic variants have a reduced bronchodilator response [54]. However, another study reported that CF patients with Arg16Gly polymorphism had better spirometry, although Gln27Glu showed no effect [55]. Corvol et al. [56] were the first to report a possible association between the BclI polymorphism of the glucocorticoid receptors *(GR)* gene and the progression of lung disease in CF [56].

#### 5.1.5. Candidate Genes Encoding Ion Channels

The identification of alternative chloride channels and potassium channels involved in maintaining ionic balance and pH in the airways also provides new targets for the development of new therapy. At the molecular level, it has been shown that there are interactions between the CFTR protein and the epithelial sodium channel (ENaC). Mutations in genes encoding both ion channels (heterozygous genotype in trans) cause an abnormal ENaC–CFTR interaction associated with pulmonary manifestations of CF or bronchiectasis (CF-like disorders) [57]. Polymorphic variants of *EnaC* (*SCNN1B*, *SCNN1G*, and *TNFRSF1A*) are modifier genes in CF, as presented in a study by Stanke et al. [58]. In another study, Viel et al. [59] concluded that the polymorphism of the *EnaC* gamma and beta genes does not influence phenotypic severity in patients with CF [59]. The use of sodium channel blockers could be an effective therapy in CF. A study by Dorfman et al. [60] showed that *SLC9A3* gene encodes an ion channel that intervenes in the Na^+^/H^+^ exchange, thus influencing *P. aeruginosa* infection and lung function in children [60].

#### 5.1.6. Genes Encoding Cytoskeletal Proteins

Stanke et al. [61] identified two allelic variants of the *KRT8* gene (encoding Keratin, type II cytoskeletal 8) associated with the severity of CF lung disease and CFTR-mediated residual chloride secretion in F508del homozygotes patients, unconfirmed in the case of the *KRT18* gene. Because the mild *KRT8* allele is associated with CFTR-mediated residual chloride secretion, the KRT8/KRT18 heterodimeric intermediate filaments of the cytoskeleton are apparently an essential component for the proper targeting of CFTR to the apical membrane in epithelial cells [61].

### 5.2. Modifier Genes Related to Cystic Fibrosis Comorbidities

The main CF comorbidities are MI, CFRD, PI, osteoporosis, and nasal polyposis [7]. Their appearance in the evolution of CF patients has been intensively studied and involves the intervention of both genetic and environmental factors, as well as the interaction between them [7]. Along with the sweat test (chlorine ≥ 60 mEq/L), MI and PI were considered diagnostic criteria for CF [7,62,63]. Although, in some studies, there are cases with severe *CFTR* mutations which had residual pancreatic function, it is considered that the presence of MI and PI is correlated with severe *CFTR* mutations compared to those that cause pulmonary manifestations [7]. Certain allelic variants of the genes *ADIPOR2* (adiponectin receptor 2), *MSRA* (methionine sulfoxide reductase A), and *SLCA4* (solute carrier family 6 member 4) could be associated with the presence of MI in patients with CF [62,63]. Li et al. [41] tested the hypothesis that the same risk alleles (*SLC26A9*, *SLC9A3*, *SLC6A14*) for MI have a pleiotropic effect, being correlated with both the severity of lung disease, the age of onset of *P. aeruginosa* infection, and early exocrine PI [41]. The allelic variant rs7512462 of the *SLC26A9* gene causes both MI and pancreatic involvement and the allele rs17563161 of the *SLC9A3* gene is associated with MI and CF lung disease, while variant rs3788766 of the *SLC6A14* gene is correlated with MI, CF lung disease, and age of *P. aeruginosa* infection [2,41]. No correlation was found between mutations in genes encoding mucins (*MUC1*, *MUC2*, and *MUC5AC*) and increased risk of MI in patients with CF [63]. In pancreatitis, however, the interaction between the *CFTR* genotype and modifying genes remains uncertain [2]. It is not yet known whether mutations in the genes involved in intrapancreatic activation of trypsin (IPAT) or in the pancreatic secretion pathways (PSP) may influence the risk of chronic pancreatitis (CP) and recurrent pancreatitis (RP) in CF patients. In a study by Sofia et al. [73], a panel of eight genes involved in IPAT (*PRSS1*, *PRSS2*, *SPINK1*, *CTRC*, *CASR*, *CFTR*, *CTSB*, and *KRT8*) and 23 additional genes involved in PSP were analyzed [73]. The study concluded that a trans-heterozygous association between *CFTR* gene and the genes involved in IPAT and PSP may increase the risk of RP and CP in CF patients. Furthermore, the same study group demonstrated that mutations in several dozen genes involved in the six different pancreatic pathways are risk factors for CP and RP, emphasizing the idea that trans-heterozygous mutations of different genes are involved in the pathogenesis of idiopathic pancreatitis (IP). However, further studies will be needed to determine whether patients with trans-heterozygous mutations have a more severe form of pancreatitis, and to elucidate the pathogenic mechanism of pancreatitis in patients with multiple gene mutations and how these genes interact (epistasis) [73].

Approximately 3–5% of CF patients have liver disease (CFLD) manifested by cirrhosis and portal hypertension. Nine functional variants were studied in five risk genes: the allelic Z variant of the *SERPINA1* gene, but also variants of the *ACE*, *GSTP1*, *MBL2*, and *TGFβ1* genes [2]. Other studies revealed an association between pancreatic disease and allelic variants of the *EPHX1* (epoxide hydrolase 1), *DSP* (desmoplakin), *HLA-DQA1*, and *HLA-DQB1* (major histocompatibility complex) genes. Furthermore, variants of the genes *GPNMB* (NMB glycoprotein), *LGALS3* (galectin 3), *NCF2* (neutrophil cytosolic factor 2), *PTPN13* (protein tyrosine phosphatase, non-receptor) type 13), *RASGRP1* (RAS guanil releasing protein 1), and *SLC33A1* (soil carrier family 33 member 1) would be associated with liver disease, but these results require validation [7,72].

The occurrence of diabetes mellitus in patients with CF is due to the impaired endocrine pancreas which contributes to the severe evolution of the disease, due to multisystemic involvement. It is known that the development of diabetes is correlated with the interaction between genetic factors and environmental factors. The *TCF7L2* gene encodes a transcription factor belonging to the Wnt signaling pathway expressed in the liver, pancreas, and adipose tissue, with the function of regulating insulin production [75,76]. In particular, the allelic variant rs7903146 of the *TCF7L2* gene increases the risk of CFRD three times and decreases the age of onset by 7 years [75]. Blackman et al. [64] demonstrated in a study that the presence of allelic variants of the genes *TCF7L2*, *IGF2BP2* (insulin-like growth factor 2 mRNA binding protein 2), *SLC26A9*, *CDKN2A/B* (cyclin-dependent kinase inhibitor 2A and cyclin-dependent kinase inhibitor 2B), and *CDKAL1* (CDK5 regulatory subunit-associated protein 1 like 1) have been corelated with the occurrence of CFRD [64].

Allelic variants rs7817 and rs3807213 of the *IFRD1* gene are correlated with nasal polyposis in patients with CF [109]. Various studies have shown an increased incidence of lower respiratory tract infections and nasosinusal manifestations in patients with certain allelic variants of the *T2R38* gene, including those with CF [110].

Many of the initial studies of candidate genes included a small number of cases analyzed and did not include a validation cohort, producing somewhat contradictory results [85,111]. Wright et al. [112] used a strategy to identify new genes that modify the CF lung phenotype based on the analysis of a high-resolution microarray to detect single nucleotide polymorphism (SNP), and nasal respiratory epithelial cells were analyzed to investigate the molecular basis of phenotypic differences in the severity of CF lung disease [110,112].

Differences in gene expression were identified in individuals with the F508del homozygous genotype who showed the most severe lung phenotype (FEV1 below the 20th percentile) and F508del homozygous individuals, but who had the mildest form of lung disease (FEV1 above the 20th percentile). The study included 12 patients with CF of similar ages, and the clinical trials for inclusion in the study were designed to minimize environmental influences on the severity of lung disease [111]. The study also included a control group of 11 individuals without CF. Of the 11,867 genes present and identified, in 75% of the analyzed samples, a number of 652 genes had a differentiated expression according to phenotype, most in severe form of the disease (569 genes). Analysis of these genes with differentiated expression, correlated with the severity of lung disease demonstrated a significant up-regulation in severe forms of CF, of genes involved in protein ubiquitination (*p* < 0.04), mitochondrial oxidoreductase activity (*p* < 0.01), and lipid metabolism (*p* < 0.03) [111].

Studies of modifier genes in CF based on microraray analysis have important limitations, correlated with the high cost of genetic analysis, small sample size analyzed, the need to update data according to the classification of *CFTR* gene mutations, and the need for long-term monitoring of patients with CF lung disease, in order to appreciate the association with modifier genes. The identification of some functional allelic polymorphisms associated with the variable phenotype in CF requires further confirmation of association by population studies [75,111].

## 6. Whole Exome Sequencing (WES) and Genome-Wide Association Studies (GWAS)

As technology has advanced, the specific approach based on candidate gene studies has been replaced, and research has focused on analisys of the entire exome (coding sequences responsible for protein synthesis) by whole exome sequencing (WES) or the entire genome by genome-wide association studies (GWAS) [85,111]

### 6.1. Whole Exome Sequencing (WES)

Analysis of modifier genes using WES has the disadvantage of requiring the inclusion of many samples to validate the study [69]. It was proposed to compare the results in patients with phenotypes at the extremities of the spectrum of phenotypic manifestations characteristic of CF. Two of the studies that used WES identified genes that change the risk of *P. aeruginosa* infection in patients with CF lung disease [69]. Edmond et al. [113,114] showed that mutations in *DCTN4* (subunit 4 of dinactin) and the *TMC6* gene (transmembrane channel-like protein 6) are associated with an early onset of *P. aeruginosa* infection and a severe decrease in FEV1, while mutations in the *CAV2* gene (Caveolin 2), on the contrary, have a protective effect [113,114]. Another study investigating the role of *DCNT4* gene suggested an increased risk of *P. aeruginosa* infection in a subgroup of men who had two *CFTR* mutations class II [31].

### 6.2. Genome-Wide Associations Studies (GWASs)

GWASs investigate single nucleotide polymorphism (SNP) and have provided new information on the role of modifier genes in CF patients phenotype. A strong point of GWASs compared to previous studies based on the analysis of candidate genes is a significantly higher number of subjects analyzed. Three major GWASs have been published to date that have identified a number of polymorphic variants associated with different CF phenotypes [2,97,98].

Corvol’s meta-analysis, which brought together GWAS results from North America and France, included data from SNP polymorphism using microarrays analysis for 6356 CF patients, compared to the largest candidate gene-based study, which included an initial cohort of 808 patients, with replication in 498 patients [69]. In this study, FEV1 was used as a marker for the severity of lung disease. FEV1 is a clinically valuable measure of lung function and a known predictor of survival in CF [97]. However, a disadvantage would be related to the limitation of the use of FEV1 as a marker of disease severity, as a comparison between age groups, because FEV1 decreases with age [69,97]. Despite this, GWAS brought important new information [97].

### 6.3. Genome-Wide Associations Studies (GWAS) Results for Cystic Fibrosis Lung Disease

Due to the importance of pulmonary diseases in the evolution and prognosis of CF patients, the identification of genetic modifiers that influence the progression of lung disease has been the central element of GWAS. To increase the importance of GWAS, it was necessary to harmonize the lung phenotype at the level of the analyzed cohorts. Using hulich normal residual mortality adjusted for CF-related mortality (KNoRMA) as a quantitative phenotype, the International Cystic Fibrosis Gene Modifier Consortium (GMC) identified five significant regions associated with the severity of lung disease. According to this study, all five regions contain genes of interest, which most likely play a role in the variation of the lung phenotype [69,97].

To date, ongoing research has not directly indicated a mechanism of action for polymorphisms present in any of the critical regions analyzed. Understanding the mechanisms of action of polymorphisms present in these five critical regions on the pulmonary pathophysiology in CF will be an important step in the study of CF modifier genes. Global efforts to map regulatory regions, decipher the structure and function of genes (sequencing), and create databases, as well as the application of new statistical methods, will facilitate the acquisition of new information in the future [69,82].

The 6p21.3 chromosomal region contains genes that encode the human leukocyte antigen (HLA) class II antigens and regulate the immune response via antigen-presenting T cells. In a study that included 745 patients with CF and the homozygous genotype F508del, lymphoblastic cell analysis demonstrated the association between HLA class II polymorphism and age of onset, as well as the persistence of *P. aeruginosa* infection [30]. This association has been demonstrated in other non-CF lung conditions, such as asthma and increased susceptibility to allergic pulmonary aspergillosis (ABPA) [32,115].

The 5p15.33 chromosomal region contains locus of *SLC9A3* gene family (solute carrier family 9A3). Mutations in the *SLC9A3* gene have a pleiotropic effect, being associated with both the increased incidence of *P. aeruginosa* lung infection in pediatric CF patients, MI, and early-onset exocrine pancreatic disease [2,41]. Knowledge of the pleiotropic effect of modifier genes could be the basis for the development of new therapies with action at the target organs [67].

The Xq22–23 chromosomal region contains locus of the *AGTR2* gene (angiotensin II receptor type 2) and the *SLC6A14* gene family (solute carrier family 6A14). The *AGTR2* gene has many functions related to the functioning of the lungs, its mutations being correlated with the development of pulmonary fibrosis, the expression of nitric oxide synthetase (NOS) and the inflammatory process of the lungs. There are no studies that specifically analyze the role of *AGTR2* [69]. Allelic variants of the *SLC6A14* gene (which encodes an amino acid transporter) have a pleiotropic effect, which are associated with an increased risk of MI, severe lung damage, and early onset of *P. aeruginosa* infection in CF patients [41,69,110].

The 3q29 chromosomal region contains the loci of the *MUC4* and *MUC20* genes that encode mucins, i.e., proteins that contribute to the achievement of an osmotic barrier, thus having a major role in eliminating mucociliary secretions. Mutations in *MUC* genes are correlated with stasis of mucociliary secretions that are predisposed to an increased risk of respiratory infections. To date, only studies of candidate *MUC* genes have been performed [41].

The 11p12–p23 chromosomal region contains locus of the *EHF* gene (which encodes ETS homologous factor) and the *APIP* gene (which encodes the protein that interacts with Apaf-1). APIP is a methionine salvage enzyme that encodes the protein that interacts with Apaf-1; its proven role is to prevent apoptosis in the presence of hypoxia, as well as the role in the inflammatory response [77]. The EHF transcription factor has been found to be involved in F508del processing and plays a role in lesion repair and tight epithelial junction control [78]. The association between certain loci on chromosome 11 and lung disease in F508del homozygous patients was initially demonstrated in a GWAS combined with a genetic linkage study [73]. The results were subsequently reconfirmed in a European cohort [69,77].

### 6.4. Genome-Wide Associations Studies (GWAS) Results for Cystic Fibrosis-Related Diabets (CFRD)

The role of the *TCF7L2* gene in the onset of CFRD was initially demonstrated in a family-based association study. The allelic variant rs7903146 (*TCF7L2*) increases the risk of diabetes by three times and decreases the age of onset by 7 years [75]. GWAS studies, which included a large number of CF patients, appear to confirm the association with the *TCF7L2* gene and CFRD [64,74]. Data from the literature have shown that CFRD and type 2 diabetes mellitus (T2DM) have overlapping etiology and pathophysiological mechanisms, mainly represented by impaired pancreatic β cells with decreased insulin secretion, rather than decreased insulin sensitivity.

The idea that susceptibility genes for T2DM in the general population would also be risk genes for CFRD was demonstrated by comparing statistical data on T2DM risk factors (data available from the National Human Genome Research Institute–European Bioinformatics Institute catalog of published GWASs (http://www.ebi.ac.uk/gwas, accessed on 19 November 2021) [85]. Two CFRD-specific risk loci (SNPs in the *SLC26A9* and *PTMA* genes) and sixteen risk loci associated with both CFRD and T2DM were identified (e.g., *TCF7L2* and *CDKAL1*) [68].

The allelic variant rs7512462 of the *SLC26A9* gene has a pleiotropic effect and is associated with both pancreatic disease and MI, while variant rs3788766 (*SLC6A14*) is correlated with MI, lung disease, and the age of onset of *P. aeruginosa* infection [2,41].

### 6.5. Genome-Wide Associations Studies (GWAS) Results for Meconium Ileus

It is known that MI occurs more frequently in patients with CF caused by severe alleles (G542X and Phe508del) than in those with G551D alleles [116]. Beyond this, heritability studies have indicated a predominant role for non-CFTR genetic factors in the production of MI. Initial studies indicated that several genes and regions are associated with MI, but a strong association between a causative modifier gene and MI has not yet been discovered [80]. Rozmahel et al. [117] were the first to report a locus modifier for MI, named cystic fibrosis modulator locus 1 (Cfm1), located on chromosome 7 in CF-mice [117]. Heterogeneity in the 19q13.2–13.4 region, which corresponds to the Cfm1 in mice, has been associated with the presence of MI in CF patients [65].

Linkage analysis coupled with candidate gene studies have identified two genes of interest for the development of MI: *ADIPOR2* and *SLC4A4* [117]. It has also confirmed an association of the *Cfm1* gene with the occurrence of MI in CFTR-deficient mice [118,119]. The role of allelic variants of the *MSRA* gene (located on chromosome 8) in the production of MI has been proven, and the results have been validated in a family-based association study [61,65] and in an animal-based study [66].

Based on the hypothesis that certain constituents of the CFTR-associated apical plasma membrane are involved in the appearance of MI, a GWAS study that analyzed 6770 patients identified *SLC26A9* and *SLC6A14*, two new loci occupied by *ATP12A* and *PRSS1* genes, in addition to the already known susceptibility genes [71]. Data were found to suggest that these loci may affect intestinal obstruction by regulating pancreatic genes, which is evidence that the normal functioning of pancreatic enzymes in embryonic life is critical for the development of fetal intestinal cells [71].

## 7. CF Lung Disease Sverity: Non-Genetic Modifiers

Improving the survival of CF patients in the last 5–6 decades is correlated with environmental factors and cannot be attributed to modifier genes. The creation of multidisciplinary centers for the care of CF patients, the increased compliance with the prescribed treatment, and, last but not least, the new classes of drugs used for treatment have significantly contributed to the increase in life expectancy in CF. Low treatment compliance had a negative effect on lung function. Although most studies have focused on the role of genetic factors (modifier genes), which, together with *CFTR* mutations, can influence the severity of clinical manifestations, the intervention of environmental factors should not be neglected. Correlated with poor socio-economic status, exposure to cigarette smoke (including in utero) [120], polluted air, climate, and proximity to water areas (increased risk of *P. aeruginosa* infection) have been shown to have a negative impact on the prognosis in CF patients [121]. All these “modifier factors” are ecological factors. Avoiding exposure to environmental factors is an effective prophylaxis measure. For example, eliminating passive smoking for CF patients is a relatively simple measure to improve the prognosis [120]. The nutritional status of the patients also plays an important role in the evolution of the CF patients, thus having a postive effect on nutritional status [122]. Patient response to nutritional treatment varies, suggesting possible intervention of other genetic and environmental factors. Pulmonary colonization with *P. aeruginosa* and *Burkholderia cepacia* is an environmental mediated event and is associated with reduced lifespan in CF. In this sense, an important role is played by epidemiological control and prevention of the spread of infections [121,123].

## 8. Could Modifier Genes Influence the Response to CFTR Modulators?

The use of recent therapies with CFTR modulators (HEMT) has led to improved life expectancy of CF patients, leading to a significant decrease in episodes of pulmonary exacerbation, with reduced hospitalization periods and improved nutritional status [124]. CFTR modulators are small, systemically administered molecules that act either by correcting CFTR folding errors (e.g., F508del) or by restoring cAMP-dependent CFTR function (e.g., G551D). It has recently been shown that the triple association between two correctors (Tezacaftor and Elexacaftor) and a potentiator (Ivacaftor) resulted in a 14% improvement in FEV1 in patients with a single F508del mutant allele [125]. This combination of drugs is indicated in CF patients over 12 years of age who have one or two F508del mutations. These drugs also affect circulating inflammatory cells. In patients with the G551D genotype, Ivacaftor decreases the rate of loss of lung function and the rate of chronic *P. aeruginosa* infection [126].

Given the effect of modulator therapy on the evolution of CF patients, the question arose of how modifying factors could influence the response to therapeutic molecules. Data also suggest that some modifiers may effectively influence the response to modulator therapy, depending on the type of drug administered [69]. Long-term data from Ivacaftor therapy have led to the conclusion that its efficacy is extremely high when administered as monotherapy and is less high in combination with another modulator. These aspects are probably correlated with the time of administration of the drug and the stage of the disease, which are more important than the age of the patient. There are not yet enough studies on children which evaluate the effect on decreased lung function and the rate of chronic lung infections; however, it is likely that early therapy would have a much better effect on slowing and/or preventing the decline in lung function compared to late administration when lung injury are already established [69,125].

Exocrine PI is present in 85% of CF patients, the pancreatic damage being considered irreversible. Munce et al. [127] showed evidence that in three pediatric patients Ivacaftor therapy restored exocrine pancreatic function by improving clinical and biochemical parameters [127].

Early administration of CFTR modulator therapy could influence the effect of modifying factors (genetic and non-genetic) that are associated with an increased risk of *P. aeruginosa* infection. Compared to these, patients who start modulator treatment later in the course of the disease may theoretically experience greater impacts from disease-modifying factors (genetic and non-genetic) [69].

The different response to modulator therapy in patients with the same *CFTR* mutation and disease status could be due to multiple factors, such as genetic or environmental factors that influence the drug’s ability to respond to a target tissue or organ, or other genes that influence drug metabolism or concomitantly administered drugs. Insufficient data on metabolic pathway interactions are not known. In patients receiving concomitant azole antifungals (strong inhibitors of CYP3A), the modulator dose is advisedly decreased. A single gene (allelic variant rs7512463 of *SLC26A9*) has been reported to alter the therapeutic response of a modulators, but the results are contradictory in different studies [67,69]. This could be explained on the basis of genetic differences that exist in different populations [69,128].

Among the environmental factors, smoking affects CFTR function and is associated with a reduction in the effect of dual Tezacaftor/Ivacaftor therapy [129]. In vitro, *P. aeruginosa* infection reduces *CFTR* gene expression and function and limits the correction of the F508del mutation by Lumacaftor [130].

## 9. Genetic Counseling of CF Patients in the Context of the Action of Modifier Genes

Genetic counseling in CF patients focuses on advising the patient/family on the manifestations of the disease, the evolution, the correlation between the type of *CFTR* mutation (genotype), and the clinical features (phenotype). The risk of recurrence of the disease in the family is also calculated. With the appeareance of the new concept of personalized medicine, based on the patient and not on the disease (“treat the patient not the disease”), it was necessary to extend the counseling to the indications/contraindications of new therapies with CFTR modulators (correctors and potentiators). Increasing life expectancy and quality of life will allow new studies and analysis of phenotypic variations in patients with the same genotype. Identification of polymorphisms of modifier genes could become an integral part of genetic counseling in CF patients [69].

## 10. Discussion

Identification of the *CFTR* gene and deciphering its structure and function helped to improve understanding around the pathophysiological mechanisms of the CF and the achievement of correlations between genotype and phenotype. However, based on the observation that people with the same *CFTR* gene mutation may have variable phenotypic manifestations, both in terms of the severity of pulmonary manifestations and associated comorbidities, the existence of other non-CFTR phenotypic modifiers (genetic and non-genetic) was also discussed. In the early 1990s, there was evidence that, although the *CFTR* mutation class was a fairly good predictor of pancreatic disease associated with CF, this did not prove to be true for CF lung disease [85].

The identification of an increasing number of modifier genes, correlated with new genetic study technologies, led to the conclusion that epistasis could be the main mechanism that explains phenotypic variability in most genetic diseases [2]. Another aspect to consider was that modifier genes may have their own contribution to the modification of the phenotype, but are in constant interaction with environmental factors, which may increase or decrease their effect. The impact of modifier genes identification is multiple, because understanding the contribution of different genes to phenotype can lead to improved prognosis, new therapeutic approaches, and personalized and patient-centered medicine. In our paper, we presented different types of studies used to identify loci and genes which may influence the phenotype of CF patients.

A first approach was based on possible candidate gene studies and involved knowledge of the pathogenic mechanism of the disease and selection of genes, which were studied later. The disadvantages of this type of study were correlated with the small number of samples analyzed, the lack of replication of results, as well as the contradictory results obtained in other studies [7,8,69].

Moreover, highlighting the role of genetic factors in producing the variability of the disease phenotype required family-based association studies. Parent genotyping established the mode of transmission of the disease, while studies on monozygotic twins and siblings were needed to prove or to exclude the phenotypic correlation within families and to prove the genetic model based on the variable expressivity of the disease. Considering the permanent interaction between genetic factors and environmental factors, longitudinal studies are needed to define the key factors in the gene–environment interaction in patients with monogenic diseases [2].

Belonging to a certain ethnic group plays an important role in characterizing the effect of modifier genes, because they differ by ethnicity and their complexity increases in ethnically mixed populations [69,128]. Although costly, GWASs have provided new information, significantly expanding knowledge on possible phenotype modifier genes and the mechanism by which they act in CF. A strong point of GWAS compared to candidate gene studies is the significantly higher number of samples analyzed [69,131]. GWASs obtain information related to numerous SNP polymorphisms at the level of whole genome, which can play a significant role in modifying the phenotype. Additionally, the results provided by GWASs are much more promising, both by identifying new gene variants and by highlighting variants with pleiotropic effect. GWASs also have several disadvantages, including the inability to both prove the relationship of association and causality and to interpret the significance of gene variants in a clinical context. Identifying a locus associated with the disease in one ethnic group/geographic region and its absence in another can be a challenge [131].

Detection of different risk alleles from one geographical region to another requires a study with a much larger number of samples. The combination of GWAS results with those obtained by sequencing the entire exome (WES) in studies that include large cohorts will contribute, in the near future, to the validation of previously obtained information. The analysis of the whole genome also allows the identification of genetic factors that create the predisposition for multifactorial (polygenic) diseases [68]. In the near future, the identification of modifier genes for monogenic diseases (such as CF) will allow a prediction to be made of how they will influence patients phenotypes [2].

As new classes of drugs are approved, it will be important to analyze how they respond to the therapy and the adverse effects that occur, in relation to the age of patients and time of administration. Using larger and larger cohorts, in which patient responses to pharmacological agents and exposure to environmental factors are recorded in detail, pharmacogenomic studies and gene–environment interaction will be possible. Future research will most likely succeed in bringing new information on how modifier genes can influence and modify the response to drug therapy, an aspect correlated with the type of CFTR modulator administered [2,7].

## 11. Conclusions

Although CF is a monogenic disease, people with the same pathogenic mutation may have a variable phenotype, due to the intervention of modifier genes or environmental factors. The research of modifier genes opens up new perspectives, both in terms of diagnosis and prognosis of the disease, as well as therapeutic intervention. The number of the genes that can modify the phenotype in CF is impressive, and their identification offers a new perspective on the pathophysiological mechanisms of the disease, paving the way for the understanding of other genetic diseases. In the near future, genetic analysis, such as WES or GWAS, will be performed routinely and the information provided can improve the diagnosis and prediction of the phenotype by including all genes known to be involved in the manifestations of a specific disease in the study. The identification of genetic variants of modifier genes could become an integral part of genetic consultation and counseling in CF patients. The new classes of modulators capable of restoring CFTR function will change the prognosis of patients, especially those receiving early treatment. It remains to be seen whether the modifier factors identified in untreated patients remain relevant in patients receiving modulator therapy. Because the response to CFTR modulator therapy is variable, in the future, there will be an increased interest in the factors that influence the therapeutic response. Environmental factors (such as exposure to tobacco smoke, nutritional status, and adherence to treatment) may influence the response to medication and, thus, should not be ignored.

## Figures and Tables

**Table 1:** Phenotype modifier gene studies (lung disease and associated comorbidities) in patients with cystic fibrosis.

Method CGA/ GWAS	Gene	CF Lung Disease	*P. aeruginosa* Infection	MI	PI	CFRD	CFLD	Study/Author References
CGA	*TGFβ1*	+	-	-		-	+	[8,9,10,11,12,13,14,15]
CGA	*IL8*	+	+					[16,17]
CGA	*IL1B*	+	+					[18,19]
CGA	*IL10*	+	-					[17,20,21,22]
CGA	*TNFα*	+	+					[23,24,25,26]
CGA	*8.1 AH*	+	+					[27,28,29]
GWAS	*HLA II*	+	+					[30,31]
CGA	*SERPINA1*	+					+	[2,8,9,32,33,34,35]
CGA	*AGER*	+						[8,36]
CGA	*MIF*	+	+					[37,38]
CGA /GWAS	*MUC5B* *MUC4* *MUC 20*	+						[39,40,41]
CGA	*MBL2*	+	+				+	[2,42,43]
CGA	*CD14*	+						[44,45,46,47,48]
CGA	*NOS*	+	-					[49,50,51,52]
GA	*ADRB2*	+	+	+				[53,54,55]
CGA	*GR*	+						[56]
CGA	*EnaC* *SCNN1B*, *SCNN1G* *TNFRSF1A*	+						[57,58,59,60]
CGA	*KRT8* *KRT19*	+						[61]
GWAS	*ADIPOR2*			+				[7,62,63]
GWAS	*SLCA4*			+				[60,62]
GWAS	*MSRA*			+				[60,62,64,65,66]
GWAS	*SLC26A9*			+	+			[64,67,68,69]
CGA/ GWAS	*SLC9A3*	+	+	+				[41,60]
GWAS	*SLC6A14*	+	+	+				[2,41,69,70,71]
CGA	*EPHX1* *GPNMB* *DSP*				+			[7,72]
CGA/ GWAS	*TCF7L2*					+		[64,68,73,74,75,76]
CGA/ GWAS	*IGF2BP2* *CDKN2A/B* *CDKAL1*					+		[64]
GWAS	*EHF*	+						[77,78]
GWAS	*ATP12A* *PRSS1*			+				[71,73]

CGA—candidate gene studies; GWAS—genome-wide association studies; MI—meconium ileus; PI—pancreatic insufficiency; CFRD-CF—related diabetes; CFLD-CF—associated liver disease.

**Table 2:** Class of *CFTR* gene mutations: genotype-phenotype correlations [5,69,82,83,84].

Class of Mutation	Class I	Class II	Class III	Class IV	Class V	Class VI
Severity	Severe	Severe	Severe	Mild	Mild	Mild
Type	Nonsense/ Frame-shift	Missense; amino acid deletion	Missense	Missense	Missense splicing defect	Missense
Frequent mutation	G542X, R553X, R1162X, W1282X	G85E, I507del, F508del, N1303K	S549R, G551D, G1349D	R117H, R347P, R334W, R1070W	A455E 3272-26A > G	4326del TC, Gln1412X, 4279insA
CFTR defect	No CFTR synthesis	CFTR trafficking and processing defect	Abnormal channel function, block in regulation; defecting gaiting regulation	Abnormal channel function, decreased conductance	Reduced synthesis of CFTR protein	Decreased protein stability
Potential therapy	Read-throug agents (Ataluren, amynoglicosydes)	Correctors (+Potentiators) Lumacaftor (+Ivacaftor)	Potentiators (Ivacaftor)	Potentiators (Ivacaftor)	Splicing modulators amplifiers	Stabilizers HGF (hepatocyte growth factor)

## Data Availability

Not applicable.

## Abbreviation

CF (Cystic Fybrosis); CFTR (CF Transmembrane Conductance Regulator); CGA (candidate gene study); GWAS (genome-wide association study); WES (whole exome sequencing); MI (Meconium ileus); PI (Pancreatic insufficiency); CFRD (CF-related diabetes); T2DM (type 2 diabetes mellitus); HLA (major histocompatibility complex); 18.1AH (8.1 ancestral haplotype [HLA-A1, C7, B8, C4AQ0, C4B1, DR3, DQ2)];ACE (angiotensin I converting enzyme); ADIPOQ (adiponectin, C1Q and collagen-containing domain); ADRB2 (Adrenoceptor Beta 2); AGER (advanced glycosylation end-product specific receptor); IFRD1 (interferon-related develop-mental regulator 1); IL10 (interleukin 10), IL1B (interleukin 1 b); IL8 (interleukin 8); MIF (macrophage migration inhibitory factor), SERPINA1 (serpin family A member 1); SERPINA3 (serpin family A member 3); TGFb1 (transforming growth factor b-1), TNFa (tumor necrosis factor a-like); CD14 (CD14 molecule); IFNGR1 (interferon g receptor 1), MBL2 (mannose binding lectin 2); TLRs (toll like receptors); TLR5 (toll like receptor 5), TLR9 (toll like receptor 9); GST (glutathione S-transferase); NOS1 (nitric oxide synthase 1, neuronal NOS), NOS3 (nitric oxide synthase 3, endothelial NOS); SCNN1B (sodium channel epithelial 1 subunit beta), SCNN1G (sodium channel epithelial 1 subunit 1 g); SLC9A3 (Solute Carrier Family 9 Member A3); KRT8 (keratin 8), KRT18 (keratin 18); MUC 4 (mucine4); TNSRS1A (TNF Receptor Superfamily Member 1A); SPINK1 (pancreatic secretory trypsin inhibitor); PRSS1 (protease serine 1); CASR (calcium-sensing receptor); CTRC (chymotrypsinogen C).
